# Identification of VEGF Signaling Inhibition-Induced Glomerular Injury in Rats through Site-Specific Urinary Biomarkers

**DOI:** 10.3390/ijms222312629

**Published:** 2021-11-23

**Authors:** Yi Yang, Kenneth Kowalkowski, Rita Ciurlionis, Wayne R. Buck, Keith B. Glaser, Daniel H. Albert, Eric A. G. Blomme

**Affiliations:** Global Pharmaceutical Research and Development, AbbVie, 1 North Waukegan Road, North Chicago, IL 60064, USA; Kenneth.Kowalkowski@abbvie.com (K.K.); Rita.Ciurlionis@abbvie.com (R.C.); Wayne.Buck@abbvie.com (W.R.B.); Keith.Glaser@abbvie.com (K.B.G.); Daniel.H.Albert@abbvie.com (D.H.A.); eric.blomme@abbvie.com (E.A.G.B.)

**Keywords:** vascular endothelial growth factor, glomerular injury, urinary biomarkers

## Abstract

Cancer therapies targeting the vascular endothelial growth factor (VEGF) signaling pathway can lead to renal damage by disrupting the glomerular ultrafiltration apparatus. The objective of the current study was to identify sensitive biomarkers for VEGF inhibition-induced glomerular changes in rats. Male Sprague-Dawley rats were administered an experimental VEGF receptor (VEGFR) inhibitor, ABT-123, for seven days to investigate the correlation of several biomarkers with microscopic and ultrastructural changes. Glomeruli obtained by laser capture microdissection were also subjected to gene expression analysis to investigate the underlying molecular events of VEGFR inhibition in glomerulus. ABT-123 induced characteristic glomerular ultrastructural changes in rats, including fusion of podocyte foot processes, the presence of subendothelial electron-dense deposits, and swelling and loss of fenestrations in glomerular endothelium. The subtle morphological changes cannot be detected with light microscopy or by changes in standard clinical chemistry and urinalysis. However, urinary albumin increased 44-fold as early as Day three. Urinary β2-microglobulin levels were also increased. Other urinary biomarkers that are typically associated with tubular injury were not significantly impacted. Such patterns in urinary biomarkers can provide valuable diagnostic insight to VEGF inhibition therapy-induced glomeruli injuries.

## 1. Introduction

Angiogenesis refers to the development of new blood vessels through capillary sprouting from pre-existing vessels. It is an essential process in normal as well as diseased states, especially in oncology since it promotes tumor growth and metastasis. Angiogenesis is controlled by a balance of pro- and anti-angiogenics factors. Among the pro-angiogenic factors, members of the vascular endothelial growth factor (VEGF) play a central role in tumor angiogenesis by stimulating the proliferation and migration of endothelial cells. There are five mammalian members of the VEGF family: VEGF-A, placental growth factor (PlGF), VEGF-B, VEGF-C, and VEGF-D, and three receptors have been identified: VEGFR-1/Flt-1, VEGFR-2/Flk-1/KDR, and VEGFR-3/Flt-4 [1].

Since increased VEGF levels correlates with tumor aggressiveness, metastatic potential and relapse, inhibition of the VEGF signaling pathway, either by blocking the extracellular bindings of VEGF to its receptor (antibodies to VEGF or extracellular domains of VEGF receptors) or by inhibiting the activation of VEGFRs (receptor tyrosine kinase inhibitors, a.k.a RTK inhibitors), has become an established therapy for the treatment of many types of tumors in the clinic [2]. However, although VEGF signaling is critical for tumor angiogenesis, normal tissues also require VEGF for vascular homeostasis, especially for blood vessels lined by fenestrated endothelial cells [1]. Particularly in the kidney, VEGF is highly expressed in normal podocyte foot processes and VEGFRs are expressed primarily in glomerular endothelial cells, as well as by mesangial cells and peritubular capillaries [2,3]. This expression pattern is not unexpected given that this tissue is highly vascularized, and it correlates with an important role in maintaining the normal glomerular filtration barrier. Hence, not surprisingly, drugs targeting the VEGF/VEGFRs axis can lead to renal damage. For example, in mice, inhibition of the VEGF signaling pathway, either by pharmacological agents or by podocyte-specific deletion of VEGF, led to pathological changes characterized by loss of endothelial fenestrations in glomerular capillaries, loss of podocytes, and proteinuria [4,5,6]. Likewise, in the clinic, proteinuria is one of the common side effects associated with VEGF inhibition therapy. Depending on the clinical trials, 21~64% of patients receiving Bevacizumab therapy (monoclonal antibody against VEGF-A, also known as Avastin) developed proteinuria and 2.1–11.9% of them were considered severe cases (grade 3–4) [2,7,8]. Similarly, proteinuria and renal insufficiency were reported in patients receiving Sunitinib (an RTK inhibitor, also known as Sutent) [9]. This impact on renal function may result in discontinuation of VEGF inhibition therapy. For example, it is recommended to suspend bevacizumab temporarily for proteinuria ≥ 2 g/24 h and to discontinue bevacizumab for grade 4 proteinuria [10].

In patients receiving VEGF inhibition therapy, proteinuria is closely monitored as an indicator of the structural damage to glomeruli. However, the level of proteinuria may not directly correlate with the severity of VEGF inhibition-induced glomerular changes. Among patients with the most severe histopathological changes as diagnosed by renal biopsy, more than 50% of them presented only mild proteinuria (grade 1–2) [11]. On the other hand, although grade 3 and 4 proteinuria reflects glomerular injury, milder grades of proteinuria may be caused by glomerular or tubular defects [12], and the latter is unlikely to be caused by VEGF inhibition. Therefore, since treatment options and prognosis vary according to the actual histological diagnosis, patients with proteinuria before or under VEGF inhibitors often need additional evaluations, such as renal ultrasound and renal biopsy.

The “omics” technologies have been very useful tools for the discovery of novel biomarkers for kidney injury in animal models [13,14]. Some of these biomarkers, especially urinary, have been evaluated for their performance characteristics in rats through large-scale collaborative efforts and have been qualified in the context of nonclinical drug development by regulatory authorities in the USA, Europe, and Japan. In clinical studies, increased levels of such biomarkers have also been observed in patients with acute kidney injuries or chronic kidney diseases. Their utility as translational biomarkers in humans are now recognized by regulatory agencies, and pharmaceutical developers have been using them in their nonclinical safety assessments. Compared to the conventional biomarkers of renal injury, such as blood urea nitrogen (BUN) or serum creatinine (sCreat), these urinary biomarkers demonstrate increased sensitivity and specificity in detecting drug-induced renal injuries in preclinical species and can inform on the injury site along the nephron [15,16,17,18,19,20]. For example, biomarkers, such as glutathione S-transferase alpha (GSTα), glutathione S-transferase mu (GSTμ or GSTYb1), and renal papillary antigen-1 (RPA1), are constitutively expressed in different parts of the kidney and are leaked into the urine from the injured cells expressing them [16]. Others, such as kidney injury molecule-1 (KIM1), neutrophil gelatinase-associated lipocalin (NGAL), osteopontin (OPN), clusterin (CLU), and trefoil factor 3 (TFF3), are significantly up-regulated (or down-regulated for TFF3) and released into the urine in response to proximal tubule injuries [17,18,19,20]. Finally, increases of urinary albumin (ALB) or β2-microglobulin (B2M) levels can reflect dysfunction in the glomerular filtration apparatus and/or impairment or saturation of the tubular re-absorption machinery [17,18].

The objective of the current study was to assess the performance of several urinary biomarkers in detecting VEGF inhibition-induced glomerular changes in rats. Male Sprague-Dawley rats were administered an experimental VEGFR inhibitor, ABT-123, for seven days to induce glomerular changes without injuries to other parts of the nephron. A battery of biomarkers was quantified at the protein level in urine and at the mRNA level in dissected glomeruli in the context of the microscopic and ultrastructural changes to decipher the mechanism of VEGF inhibition-induced glomerular injury.

## 2. Results

### 2.1. Pharmacological Profile of ABT-123

**Figure 1:**
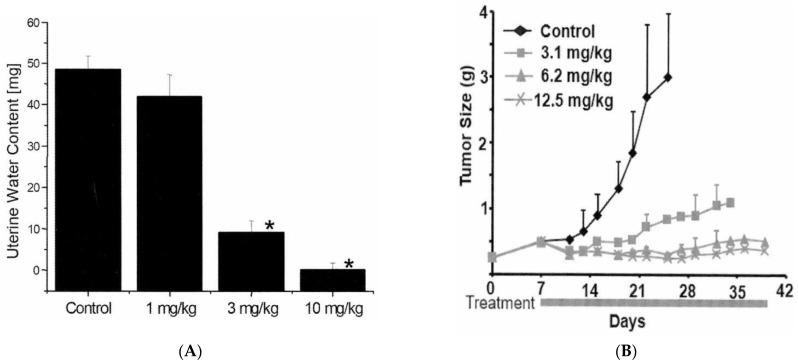
In vivo pharmacology of ABT-123. (**A**) Inhibition of uterine edema in mice by ABT-123. Mice (*n* = six per group) were orally administered ABT-123 or vehicle 30 min prior to an i.p. injection of 25 μg estradiol. The uterus was removed 2.5 h following estradiol stimulation. The uterine water content was expressed as the difference between wet and blotted uterine weights. ABT-123 induced a statistically significant decrease (* *p* < 0.01) in mean water content at 3 and 10 mg/kg. (**B**) Effects of ABT-123 on the growth of HT1080 human tumor cells implanted subcutaneously in the flank of severe combined immunodeficiency disorder mice. Mice (*n* = ten per group) were dosed with ABT-123 or vehicle daily starting on Day seven. Tumor volumes are expressed as mean ± SD. Statistically significant differences (*p* < 0.05) in mean tumor volume were observed for all treatment groups by Day fourteen.

ABT-123 is a potent and selective inhibitor of VEGFRs, with IC50 values against VEGFR-1, -2, and -3 of 70, 24, and 54 nM, respectively. There was over 150-fold selectivity against non-related tyrosine kinases or serine/threonine kinases (Table 1). In the rat uterine edema model, estradiol stimulation increased vascular permeability through upregulation of VEGF, resulting in increased water content in uterus (i.e., edema) [21]. ABT-123 dose-dependently reduced the estradiol-induced edema in this model with an estimated ED50 of 2 mg/kg (Figure 1A). In the HT1080 human fibrosarcoma xenograft model, ABT-123 resulted in dose-dependent and potent inhibition of tumor growth with an estimated ED75 of 4 mg/kg (Figure 1B).

### 2.2. Toxicity Profile of ABT-123 in Rats

In a seven-day repeated dosing study in male SD rats (three/group), ABT-123 was well-tolerated at 1 and 3 mg/kg with no clinical observations and no significant changes in body weight and food consumption. At 10 mg/kg, ABT-123 caused dehydration, decreased food consumption, and body weight loss (5–10% lower than baseline body weights). One rat at 10 mg/kg was found dead on Day eight just before sacrifice.

**Figure 2:**
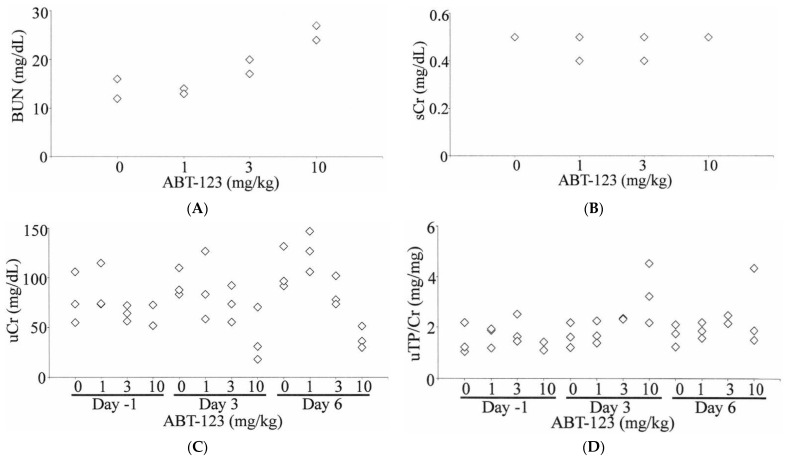
Effects of ABT-123 on conventional kidney injury biomarkers in rats. Male SD rats were administered with ABT-123 at 0, 1, 3, and 10 mg/kg/day once daily for seven days. BUN (**A**) and serum creatinine (**B**) were evaluated on Day 8 at necropsy (24 h after the last dose). Urinary creatine (**C**) and total protein (**D**) were measured at baseline, Day three, and Day six. Each diamond represents an individual animal.

Mild increases of BUN (up to two-fold relative to vehicle control) were observed in one rat dosed at 3 mg/kg and two rats dosed at 10 mg/kg (Figure 2A). However, there were no changes in sCr levels (Figure 2B). The urine volumes collected on Day three and Day six from animals at 10 mg/kg were approximately two-fold higher than those in the vehicle control group. The changes in urine volumes were associated with a 50% decrease of uCr levels (Figure 2C). Therefore, there were no significant changes in the total amount of creatinine excreted in the urine in any of the rats at any time point. When normalized to uCr levels, a 2.5-fold increase of uTP was observed in one rat dosed at 10 mg/kg as early as Day three (Figure 2D). This rat was later found dead on Day 8 just before the scheduled necropsy.

Other clinical pathological changes included dose-dependent increases of ALT, AST, RBC, and Hct levels at ≥1 mg/kg (Supplemental Material). Rats at 10 mg/kg also showed increases of sodium and chloride, and decreases of total protein, potassium, calcium, phosphate, glucose, platelets, and reticulocytes (Supplemental Material).

**Figure 3:**
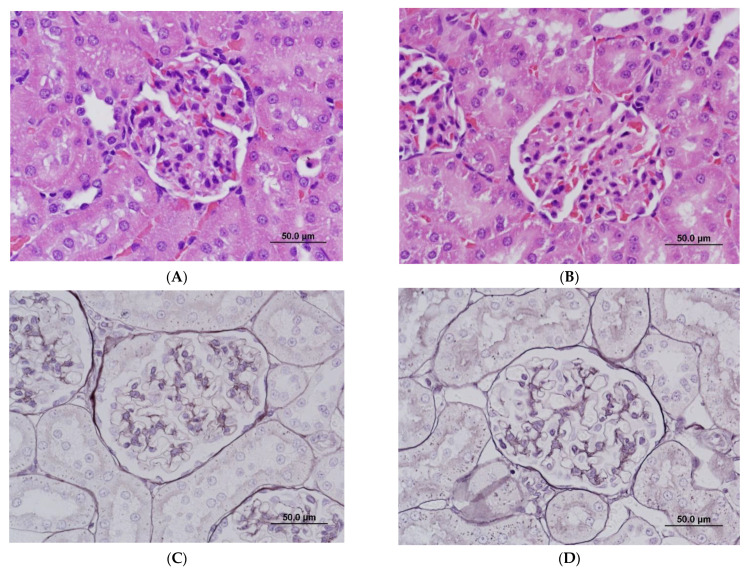
Light microscopy of glomeruli in rats treated with vehicle control (**A**,**C**) and ABT-123 at 10 mg/kg/day (**B**,**D**) for seven days. No significant light microscopic changes are apparent on hematoxylin and eosin (**A**,**B**) and Gomori methenamine silver (**C**,**D**) preparations. Scale bar = 50 μm.

At the light microscopic level, there were no significant changes in kidneys. At 10 mg/kg, changes in the thickness of the glomerular mesangium ranged from minimal to undetectable and were considered within the range of normal variation (Figure 3B). Gomori methenamine silver staining of basement membranes did not detect duplication or splitting of glomerular basement membranes (Figure 3D). Non-kidney histopathological changes were observed at 10 mg/kg and included mild hepatocellular apoptosis, cellular infiltrates and/or epithelial hyperplasia in the non-glandular stomach and gastrointestinal tract, apoptosis in the pancreas, and hypocellularity in the spleen, thymus, and bone marrow.

### 2.3. Glomerular Ultrastructural Changes in Rats Treated with ABT-123

**Figure 4:**
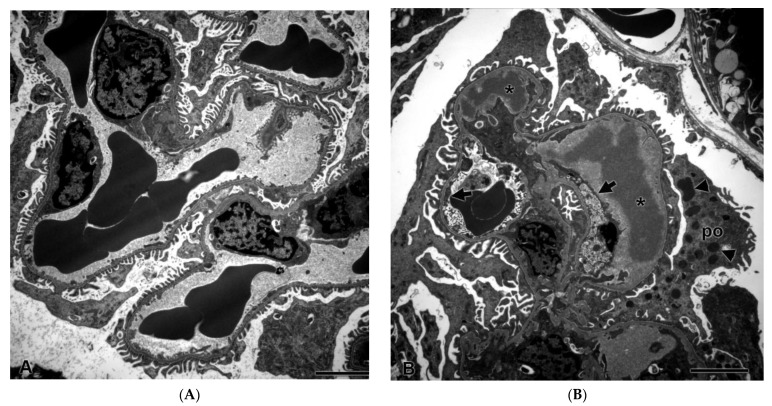
Glomerular ultrastructural changes in rats treated with vehicle control (**A**) and ABT-123 at 10 mg/kg/day (**B**) for seven days. Rats treated with ABT-123 showed irregularly shaped foot processes of podocytes (po), loss of fenestrations in endothelium (arrow), and electron-dense deposits (stars) or granules (triangle) in subendothelial space and in podocytes, respectively. Scale bar = 2 μm.

Despite a lack of histopathological changes in the kidney under light microscopy, there were ultrastructural changes in glomeruli in all three rats after seven days of dosing with ABT-123 at 10 mg/kg. As shown in Figure 4B, glomerular endothelial cells were swollen with loss of fenestrations and there was a loss of the endothelial cell lining in some glomerular capillary loops. There were subendothelial electron-dense deposits, consistent with proteinaceous material in the glomerular basement membrane. Podocytes exhibited striking alterations, including fusion of foot processes and the presence of large numbers of cytoplasmic electron-dense granules that were consistent with proteinaceous material endocytosed from the glomerular filtrate. There were no ultrastructural changes in rats from the lower dose groups.

### 2.4. Urinary Biomarker Changes in Rats Treated with ABT-123

**Figure 5:**
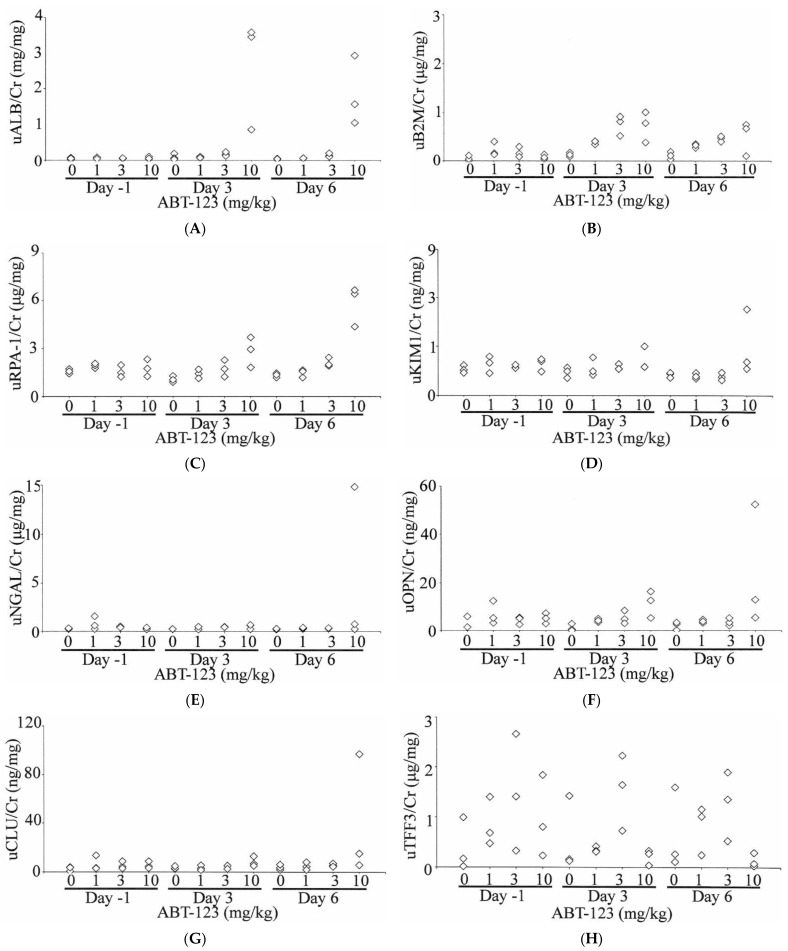

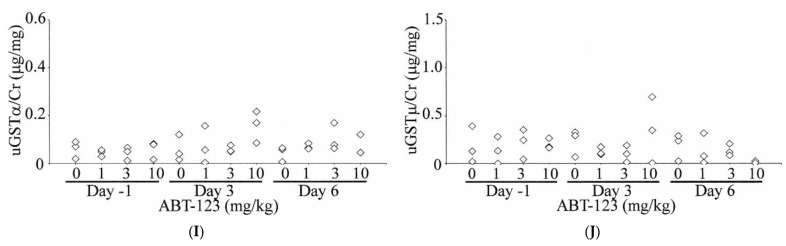
Effect on ABT-123 on urinary biomarkers in rats. Male SD rats were administered with ABT-123 at 0, 1, 3, and 10 mg/kg/day once daily for seven days. Urinary albumin (**A**), β2-microglobulin (**B**), RPA-1 (**C**), KIM1 (**D**), NGAL (**E**), osteopontin (**F**), clusterin (**G**), trefoil factor family 3 (**H**), GSTα (**I**), and GSTμ (**J**) were measured at baseline, Day three, and Day six. Each diamond represents an individual animal. Values were normalized to the concurrent urinary creatinine concentration.

A total of 10 urinary biomarkers, including ALB, B2M, RPA-1, KIM1, NGAL, OPN, CLU, TFF3, GSTα, and GSTμ, were evaluated in rats treated with ABT-123 at pre-dose (Day -1) and on Days three and six post-dose. As shown in Figure 5, urinary ALB levels were increased compared to baseline values as early as Day three in all three rats treated with ABT-123 at 10 mg/kg (11- to 44-fold on Day three and 13- to 36-fold on Day six relative to the upper limit of normal range). B2M levels were also increased (up to six-fold) in two animals administered ABT-123 at 1 mg/kg on Day three and two animals administered ABT-123 at 10 mg/kg on Days three and six. Two animals at 10 mg/kg also showed four-fold increase of RPA-1 levels on Day six. In contrast, there were no changes in the levels of KIM1, NGAL, OPN, and CLU except on Day six in one rat at 10 mg/kg, which survived until scheduled necropsy. GSTα and GSTμ levels were variable among rats with no clear trend for all doses and time points. When compared to time-matched vehicle controls, TFF3 levels were generally lower in rats at 10 mg/kg. However, there was significant variability in TFF3 levels even among control animals. When TFF3 levels were normalized to the respective rat’s baseline value, the rats at 10 mg/kg had over 80% reduction of TFF3 on Day six relative to Day -1.

### 2.5. Gene Expression Changes in Rats Treated with ABT-123

Overall, ABT-123 didn’t induce a significant amount of gene expression changes (up to 3%) at any dose levels either in kidney samples or in glomerular samples. Table 2 showed the relative gene expression changes of the urinary biomarkers in kidney and more specifically, in glomeruli, from rats treated with ABT-123 at 10 mg/kg. Among the genes representing the 10 urinary biomarkers, Tff3 was significantly down-regulated in glomerular samples, but was not significantly changed in kidney samples. Opn and Gstμ were up-regulated in both kidney and glomerular samples. Kim1, Ngal, and Clu were upregulated in kidney samples, but were not significantly changed in glomerular samples. There were no significant gene expression changes in any of these biomarkers at 1 and 3 mg/kg.

Among the Vegf receptors, both Vegfr-1 and Vegfr-2 were down-regulated in glomerular samples from rats treated with ABT-123 at 10 mg/kg (Table 3). Similar trends were also observed in the kidney samples from these rats. The VEGF family genes were mostly unchanged, except that Vegfc was downregulated in the glomerular samples from one rat treated with ABT-123 at 10 mg/kg.

## 3. Discussion

In the current study, we generated an experimental rat model of VEGF inhibition-induced glomerular injury using a highly specific VEGFR inhibitor, ABT-123. At 10 mg/kg, which is a dose resulting in slightly higher systemic exposure than those associated with anti-tumor efficacy in animals, there were no histopathological changes at light microscopic level in the kidneys after seven days of treatment. However, characteristic ultrastructural changes could be detected by electron microscopy in glomerular endothelial cells and podocytes. A subset of urinary biomarkers (in particular, urinary ALB) were elevated in these rats, indicating the presence of glomerular injury.

Inhibition of the VEGF signaling pathway, either by targeting the ligand VEGF or by targeting the receptor VEGFR, is associated with nephrotoxicity characterized morphologically by glomerular damage and clinically by proteinuria [2,8]. However, accumulating evidence suggests that blocking VEGF or its receptor results in different changes in the glomerular apparatus. Specifically, targeting VEGF tends to affect glomerular endothelial cells first, while VEGFR inhibition does not appear to be limited to the endothelium [8]. In mouse models where the VEGF gene was knockout specifically in podocytes, glomerular changes were initially limited to endothelial cells with swollen endothelial cells and dense subendothelial deposits being the main finding. Podocytes were relatively well preserved at early stages before becoming abnormal as the renal toxicity progressed [6]. The specificity of the insult to the glomerular endothelial cells has also been observed in kidney biopsy samples from patients experiencing nephrotoxicity after anti-VEGF therapy: most of these patients developed renal thrombotic microangiopathy (TMA), characterized by glomerular endothelial cell swelling and focal glomerular capillary thrombosis [8]. In addition, circulating inflammatory cells were often present in the glomerular capillary lumen. In contrast, vandetanib, a VEGFR inhibitor, induced in rats not only the loss of fenestration in glomerular endothelial cells, but also morphological changes in podocytes [22]. This is consistent with observations in kidney biopsy samples from patients under treatment with VEGFR inhibitors, where minimal change nephropathy/focal segmental glomerulopathy (MCN/FSG)-like lesions were observed and where podocyte alternations were the dominant feature [8]. However, TMA-type of changes have also been reported in patients receiving VEGFR inhibitors [8,23]. In our study, ABT-123 at 10 mg/kg induced ultrastructural changes in both glomerular endothelial cells and podocytes. Electron-dense deposits, consistent with proteinaceous material, were observed in the basement membrane and in the cytoplasm of podocytes. Mesangiolysis or thrombi were not present in any rat kidney samples. Therefore, the ultrastructural changes observed in our study confirmed what has been previously reported in experimental rodent models and in patients under treatment with small molecule VEGFR inhibitors.

Mechanistically, although the VEGFR is mainly expressed in endothelial cells, VEGFR inhibition can lead to podocyte injury by promoting an abnormal endothelial-podocyte crosstalk. One hypothesis is that VEGFR inhibition can inactivate RelA, a master subunit of NF-kB in both podocytes and endothelial cells. The lack of negative regulation from RelA, in turn, led to c-mip overexpression in podocytes, which results in cytoskeletal alterations in podocytes, fusion of podocyte foot process, and subsequently proteinuria [2,24]. Alternatively, VEGFR inhibition could lead to paracrine inhibition of VEGF production in podocytes, which in turn inhibits nephrin, a key component of the slit diaphragm and compromise of the glomerular filtration barrier leading to proteinuria [25]. In our study, RelA, nephrin, and c-mip were not significantly regulated at the gene expression level in either kidney or glomerular samples. We also evaluated other genes encoding slit diaphragm-associated scaffolding proteins, such as Neph1, CD2-associated protein, cadherin 1, cadherin 3, and Fat1. Neph1 was slightly down-regulated in glomerular samples from rats treated with ABT-123 at 10 mg/kg, but was not changed in kidney samples (data not shown). There were no gene expression changes in other slit-diaphragm-related proteins in either kidney or glomerular samples. In addition, while VEGFs were not changed at the gene expression levels, both Vegfr-1 and Vegfr-2 mRNA levels were downregulated in glomeruli, which is consistent with observations in patients taking VEGFR inhibitors [24,26].

In the clinic, kidney changes associated with inhibition of VEGF signaling are monitored by measuring urinary protein levels. However, this is not a very sensitive biomarker of nephrotoxicity indued by anti-VEGF therapies. Early glomeruli injuries in patients under these therapies are typically undetected [11]. In our study, except for a two-fold elevation of BUN in a few animals, there were no significant changes in conventional kidney injury biomarkers (BUN, serum creatinine, and urinary protein) in rats treated with ABT-123 at 1, 3, and 10 mg/kg for seven days. Moreover, since these increases in BUN levels were not associated with concurrent creatinine changes, they were likely due to dehydration and thus did not necessarily reflect nephrotoxicity. Therefore, those conventional kidney injury biomarkers are not sensitive and specific enough to identify early, subclinical levels of VEGF-inhibitor induced glomerular injury, hence the motivation to interrogate the utility and performance of a large panel of novel urinary biomarkers.

Among the urinary biomarkers evaluated, urinary ALB demonstrated the most pronounced changes (up to a 44-fold increase) in rats treated with ABT-123. Under normal physiological condition, only a small fraction of intermediate-molecular-weight proteins, such as albumin, can be filtered through the glomerulus with a low glomerular sieving coefficient (ranging from 0.00062–0.0341 in rats) due to the size and charge restriction [12,27,28]. The proximal convoluted tubules then reabsorb 71%, the loop of Henle and distal tubule 23%, and collecting duct 3% of the filtered albumin, resulting in only 3% of the filtered albumin present in urine of normal rats. In the proximal tubule, such active reabsorption is facilitated by multiligand receptors such as megalin, cubulin, and FcRn [29]. Consequently, increases of urinary albumin can be a result of not only increased leakage from damaged glomeruli, but also of decreased reabsorption from impaired proximal tubules [28,30]. Another possible mechanism for increased urinary albumin is related to expression of the normal silent albumin gene in proximal tubules, as observed during acute kidney injury [31]. In the current study, there were no albumin gene expression changes either in whole kidney or glomeruli samples. Furthermore, no histopathological changes were observed in kidney tubules, and there were no gene expression changes in megalin, cubulin, and FcR in kidney, suggesting the albumin reabsorption rate by proximal tubules was unchanged. Conversely, ABT-123 induced ultrastructural changes in all three layers of glomerular filtration barrier: swollen endothelial cells with loss of fenestrations, electron-dense deposits in the basement membrane, and fused podocyte foot processes. Therefore, the high levels of urinary albumin in ABT-123-treated rats were most likely due to the increased filtration from damaged glomeruli, while the reabsorption rate by proximal tubules was unchanged.

This conclusion is also supported by the lack of changes in other urinary biomarkers (KIM1, NGAL, OPN, CLU, GSTα, and GSTμ) that are typically associated with tubular injury. In the current study, only one animal at 10 mg/kg showed an increase of KIM1, NGAL, OPN, and CLU in urine. At gene expression levels, these four genes were up-regulated in kidney samples from rats treated with ABT-123 at 10 mg/kg. Except Opn, the upregulation was not present in glomeruli samples, demonstrating the site-specificity of these biomarkers. While morphologically, the ABT-123 induced kidney changes were limited to glomeruli, the small changes of these tubular injury biomarkers at the gene expression level and the urinary protein level may also suggest some sub-morphologic changes in tubular function.

Small increases of urinary B2M (up to six-fold) were also observed in a few animals treated with ABT-123 at 1 mg/kg on Day three and at 10 mg/kg on Days three and six. B2M is a polypeptide smaller than albumin with a glomerular sieving coefficient close to 1. It can freely filter through the glomerular filtration barrier, although it is almost completely reabsorbed and metabolized by proximal tubules under normal conditions [32]. Hence, increases of urinary B2M can be attributed to impairment of glomerular filtration or of tubular reabsorption capability [33,34]. In the current study, given the lack of changes in kidney tubules, it is most likely that glomerular injury induced by ABT-123 lead to higher B2M filtration, exceeding the normal tubular reabsorption capability.

TFF3 is a peptide hormone secreted by epithelial cells and functions to maintain and restore epithelial integrity. It is highly expressed in normal rat kidney. In rats, urinary TFF3 levels decreases after administration of compounds that induce acute tubular injury [18]. In our study, urinary TFF3 levels were significantly reduced on Day six in rats treated with ABT-123 at 10 mg/kg, especially when normalized to the respective baseline levels. In addition, the Tff3 mRNA was consistently down-regulated in glomeruli samples, but not in kidney samples. The data suggest urinary TFF3 may be a useful biomarker of glomerular injury.

In summary, we characterized a VEGFR inhibitor-induced glomeruli injury model in rats using a highly specific experimental VEGFR inhibitor. The ultrastructural changes in glomeruli resembled the ones observed in biopsy samples from patients receiving VEGFR therapy. The subtle morphological changes could not be detected by changes of standard clinical chemistry and urinalysis parameters. However, a significant increase of urinary albumin and the lack of changes of urinary biomarkers specific for tubular injury (such as KIM1) provided valuable diagnostic insight that the changes were specific to glomeruli. One should also note that additional research is needed to generalize these findings, given the small sample size in this study. This can be accomplished in a follow-up study with a higher number of animals and/or studies with additional structurally distinct VEGFR inhibitors.

## 4. Materials and Methods

### 4.1. In Vitro and In Vivo Pharmacology

The inhibitory effect of ABT-123 on VEGFRs and several structurally related kinases were evaluated in a high throughput screening assay using homogeneous time-resolved fluorescence technology as described by Dai, et al. [35]. Briefly, ABT-123 was added to 40 μL reaction mixture containing the kinase of interest, a biotinylated peptide substate (Biotin-Ahx-AEEEYFFLFA-amide at 4 uM), 1 mM APT (or 5 μM for serine/threonine kinases), 50 mM Hepes/NaOH (pH 7.5), 10 mM MgCl_2_, 2 mM MnCl_2_, 2.5 mM DTT, 0.1 mM orthovanadate, and 0.01% bovine serum albumin (BSA). The reaction was stopped with 10 μL/well of 0.5 M EDTA after 1 h incubation at room temperature. The time-resolved fluorescence was recorded from 1 to 4 h after addition of the detection reagent containing streptavidin-allophycocyanin (Prozyme) (1.1 μg/mL) and PT66 antibody europium cryptate (Cis-Bio) (0.1 μg/mL). Each IC50 determination was performed with seven concentrations, and each assay point was determined in duplicate.

The effect of ABT-123 on vascular permeability was evaluated in an estradiol-induced rat uterine edema model [35]. Twelve-week-old balb/c female mice (Taconic, Germantown, NY, USA) were pretreated with 10 units of pregnant mare’s serum gonadotropin intraperitoneally (i.p.) 72 and 24 h prior to estradiol. ABT-123 at the indicated dose or vehicle control were then administered 30 min prior to a bolus i.p. injection of 25 µg estradiol. The uteri were harvested 2.5 h later. The uteri were weighed, blotted to remove water, and reweighed. The difference between wet and blotted weights represented the water content of the uterus.

The anti-tumor efficacy of ABT-123 was evaluated in an HT1080 tumor growth inhibition model [35]. Briefly, 0.5 million HT1080 human fibrosarcoma cells (ATCC) were inoculated into the flank of SCID-beige mice. One week after inoculation, tumor-bearing animals were administered either vehicle or ABT-123 at the indicated dose. Tumor growth was assessed every two to three days by measuring tumor size and calculating tumor volume using the formula [length × width^2^]/2.

### 4.2. Animals, Treatment, and Sample Collection

Male Sprague-Dawley rats [Crl:CD^®^(SD)IGS BR] weighing approximately 250 g were obtained from Charles River Laboratories, Inc. (Portage, MI, USA). The animals were provided non-certified Harlan Teklad Global 2018 Rodent Diet (Harlan Teklad, Madison, WI, USA) and water ad libitum. Rats were housed two or three per cage for two days after receipt to aid in acclimation. Thereafter, rats were single housed in ventilated, stainless steel, wire bottom hanging cages equipped with feeders and an automatic watering system. The animals were orally dosed with vehicle (PEG-400), or ABT-123 at 1, 3, or 10 mg/kg through oral gavage for seven consecutive days (three rats/group). ABT-123 was formulated in PEG-400 to reach a concentration of 0.5, 1.5, and 5 mg/mL, respectively, so that each rat received a constant volume of 2 mL/kg/day. The first day of dosing was designated as Day one. On Day -1, Day three, and Day six, animals were placed in Tecniplast rat metabolic cages (West Chester, PA, USA) for approximately 17 hrs with access to food and water. During these periods, urine samples were collected into containers on dry ice from individual rats and stored at −80 °C until analyzed for urine chemistry, urinalysis, and urinary biomarkers. The animals were euthanized 24 hr after the last dose (Day 8) and blood samples were collected at necropsy via the abdominal caudal vena cava for clinical pathological examination. All animals were fasted overnight prior to blood collection. Kidney (left) samples were snap-frozen in liquid nitrogen for cryosections and RNA isolation. Samples of the following tissues were collected in 10% neutral buffered formalin for histopathological evaluation: liver, spleen, heart, kidney (right), bone (sternum) with marrow, thymus, lungs, and GI tract. Experiments were conducted in accordance with the Guiding Principles in the Use of Animals in Toxicology (2002) and the Guide for the Care and Use of Laboratory Animals (1996) and were approved by Abbvie local Institutional Animal Care and Use Committee (0609A01180, 1 October 2008).

### 4.3. Clinical Pathology and Histopathology Assessment

Serum clinical chemistry parameters were quantified using an Abbott Aeroset clinical chemistry analyzer (Abbott Laboratories, Abbott Park, IL, USA), complete blood counts and differential counts were generated with an Abbott CELL-DYN 3700 (Abbott Laboratories, Abbott Park, IL, USA), and reticulocytes were quantified using a Sysmex R3500 (Sysmex, Mundelein, IL, USA). The clinical pathological parameters included sodium (Na), potassium (K), chloride (Cl), CO2, calcium (Ca), phosphate (Phos), glucose (Glu), triglycerides (Trig), cholesterol (Chol), blood urea nitrogen (BUN), creatinine (sCr), total protein (sTP), alanine amino transferase (ALT), aspartate amino transferase (AST), alkaline phosphatase (AlkP), total bilirubin (TBIL), gamma glutamyltransferase (GGT), white blood cell count (WBC), lymphocyte count (LYM), red blood cell count (RBC), hematocrit (HcT), reticulocyte counts (RET), and platelet count (PLT).

Urine chemistry parameters, including creatinine (uCr), total protein (uTP), and urea, were quantified using the Aeroset Chemistry Analyzer (Abbott Laboratories, Abbott Park, IL, USA). uTP and urea values were normalized by uCr levels. Urinalysis were performed using an IRIS urinalysis workstation Model 500 (International remote imaging systems, Los Angeles, CA, USA) and included clarity, color, pH, specific gravity, bilirubin, blood, glucose, protein, ketone, and urobilinogen. Total urine volume was also recorded.

Formalin-fixed samples were routinely processed and embedded in paraffin. Paraffin sections (6 μm) were stained with hematoxylin and eosin for histopathological evaluation. Glomerular basement membranes were stained with the Accustain Silver stain (modified Gomori Mechenamine Silver staining) kit according to the kit’s instructions (HT100, Sigma-Aldrich, St Louis, MO, USA).

### 4.4. Electron Microscopic Assessment of the Kidney

Specimens of kidney collected for ultrastructural pathology were fixed by immersion in Karnovsky’s fixative (2.5% glutaraldehyde, 2% paraformaldehyde in 0.1 M Sorensen’s phosphate buffer, pH 7.2–7.4). Specimens were postfixed in 1% osmium tetroxide in 0.1M Sorensen’s phosphate buffer (pH 7.3), dehydrated in graded ethanol and propylene oxide, and embedded in Luft’s epoxy resin. Ultra-thin sections were cut, stained with 2% methanolic uranyl acetate followed by Reynolds’ lead citrate, and examined using a JEM1400 transmission electron microscope (JEOL, Peabody, MA, USA).

### 4.5. Urinary Biomarker Analysis

Urinary biomarkers were quantified using Meso-Scale Discovery (MSD) electrochemiluminescent immunoassays according to the manufacturer’s protocol (MSD, Gaithersburg, MD, USA). The kits used included the Rat Kidney Injury Panel 1 (ALB, KIM1, NGAL, OPN), the Argutus Acute Kidney Injury Panel (GSTα, GSTμ, RPA-1), and the rat clusterin test kit (CLU), the rat B2M test kit, and the rat TFF3 test kit. Signals were measured using the Sector Imager 6000 instrument (MSD, Gaithersburg, MD, USA). Concentrations of urinary biomarkers in each sample were then normalized to the concurrent uCr concentrations.

### 4.6. Laser Capture Microdissection and Microarray Analysis

For microarray analysis of glomeruli samples, kidney cryosections (8 μm thick) were prepared with a Microm HM505 cryomicrotome, mounted on glass slides, then rapidly stained and dehydrated using the Arcturus^®^ Histogene^®^ LCM Frozen Section Staining kit (Applied Biosystems, Bedford, MA, USA). Laser capture microdissection of glomeruli was performed using an Arcturus PixCell IIe instrument (Applied Biosystems). Total RNA was extracted and purified from approximately 70 micro-dissected glomeruli using the Arcturus^®^ PicoPure^®^ RNA Isolation kit (Applied Biosystems). 50 ng of total RNA was then amplified, reverse transcribed and labeled into SPIA cDNA using the WT-Ovation Pico RNA Amplification System (NuGEN Technologies Inc., San Carlos, CA, USA).

For microarray analysis of kidney samples, total RNA was purified from kidney samples (half, transverse bisected at ureter) using TRIzol (Invitrogen, Waltham. MA, USA) and chloroform extraction followed by isopropanol precipitation. Microarray analysis of kidney samples was performed using Affymetrix standard protocol. Briefly, 5 μg of total RNA was reverse transcribed into cDNA using a Superscript II Double-Strand cDNA synthesis kit (Invitrogen). Labeled cRNA was synthesized from the cDNA using the RNA Transcript Labeling Kit (ENZO Life Sciences, Farmingdale, NY, USA). Approximately 20 μg of cRNA was then fragmented at 94 °C for 35 min.

Labeled cRNA (kidney) or labeled amplified SPIA cDNA (glomeruli) was hybridized to GeneChip^®^ Rat Genome 230 2.0 Arrays (Affymetrix, Santa Clara, CA, USA) at 45 °C overnight. The arrays were then scanned using the Affymetrix GeneChip Scanner 3000.

### 4.7. Statistical Analysis

The normal range of each urinary biomarkers were calculated as 95% percentile of the mean value from treatment naïve animals, i.e., all animals on Day -1 and vehicle control animals on Days three and six. Due to the limited sample size, statistical significance of urinary biomarker changes in the treatment group was not assessed. Instead, urinary biomarker values were considered biologically meaningful if their value was above 3 times the upper limit of normal range.

For microarray analysis, scanned image and intensity files were imported into Resolver gene expression analysis software version 7.2.2 (Microsoft, Redmond, WA, USA). Gene expression ratios were built for each treatment animal versus the averaged vehicle controls using Resolver’s error model. To detect genes differently regulated between vehicle and ABT-123-treated rats, the ratio profiles were analyzed by error-weighted one-way ANOVA analysis using Rosetta Resolver. Genes with a fold change of ≥ 1.5 fold and a *p*-value < 0.05 were considered significant.

## Figures and Tables

**Table 1:** Kinase inhibition profile of ABT-123.

VEGFRs		Non-Related Tyrosine Kinases		Serine/Threonine Kinases	
Kinase	IC50 (nM)	Kinase	IC50 (nM)	Kinase	IC50 (nM)
VEGFR-1	70	SRC	>50,000	AKT	>50,000
VEGFR-2	24	IGFR	>50,000	SGK	11,000
VEGFR-3	54	INSR	>50,000	CDC2	>50,000
		LCK	13,000	PKA	>50,000
		EGFR	>50,000		
		HCK	>50,000		
		cMET	>50,000		
		LYN	>20,000		
		FYN	>50,000		
		FGR	>50,000		

**Table 2:** Gene expression changes of urinary biomarkers in glomerular and kidney samples from rats treated with ABT-123 at 10 mg/kg/day for seven days.

Sequence Code	Sequence Name	Sequence Description	Fold Change in Glomerulus		Fold Change in Kidney	
			Rat #1	Rat #2	Rat #1	Rat #2
1367555_at	Alb	Albumin	−8.15	−4.78	−2.20	−2.90
1367595_s_at	B2m	Beta-2 microglobulin	1.11	1.13	−1.06	−1.23
1387965_at	Kim1	Kidney injury molecule 1	1.10	2.19	**6.55**	**2.19**
1387011_at	Ngal	Neutrophil gelatinase-associated lipocalin	1.73	−1.20	**2.96**	**4.08**
1367581_a_at	Opn	Osteopontin	**1.82**	**2.30**	**2.79**	**2.90**
1367784_a_at	Clu	Clusterin	1.30	**1.67**	**2.80**	**1.48**
1388246_at	Clu	Clusterin	1.47	**1.96**	1.50	1.05
1387218_at	Tff3	Trefoil factor 3	**−14.96**	**−9.34**	−1.11	−1.21
1367774_at	GSTa3	Glutathione S-transferase A3	1.26	**−1.77**	−1.17	1.03
1372297_at	GSTa4	Glutathione S-transferase alpha 4	−1.50	**−4.49**	−1.29	−1.09
1386985_at	GSTm1	Glutathione S-transferase mu 1	**3.11**	**2.39**	**2.16**	**3.88**

Fold changes relative to vehicle controls. Bold: fold change of ≥ 1.5 fold and *p* value < 0.05.

**Table 3:** Gene expression changes of VEGFs or VEGFRs in glomerular and kidney samples from rats treated with ABT-123 at 10 mg/kg/day for seven days.

Sequence Code	Sequence Name	Sequence Description	Fold Change in Glomerulus		Fold Change in Kidney	
			Rat #1	Rat #2	Rat #1	Rat #2
1373807_at	Vegfa	Vascular endothelial growth factor A	−1.03	1.22	−1.00	1.14
1370081_a_at	Vegfa	Vascular endothelial growth factor A	−1.17	1.18	−1.07	1.02
1380854_at	Vegfb	Vascular endothelial growth factor B	−1.08	−6.07	−1.01	−1.31
1368463_at	Vegfc	Vascular endothelial growth factor C	−2.46	**−2.76**	−1.48	−1.25
1387709_at	Vegfd	Vascular endothelial growth factor D	1.17	1.44	−1.13	−1.15
1368919_at	Pgf	Placental growth factor	1.66	2.28	−1.17	1.20
1368918_at	Pgf	Placental growth factor	1.48	1.89	1.31	−1.29
1369087_at	Vegfr-1	Vegf receptor 1	**−2.94**	**−3.29**	**−1.60**	**−1.13**
1367948_a_at	Vegfr-2	Vegf receptor 2	**−2.51**	**−2.21**	**−3.30**	**−2.45**
1369216_a_at	Vegfr-3	Vegf receptor 3	2.10	1.15	1.10	−1.16

Fold changes relative to vehicle controls. Bold: fold change of ≥ 1.5 fold and *p* value < 0.05.

## Data Availability

Data is contained within the article and Supplementary Material.

## Supplementary Materials

All data are available online at https://www.mdpi.com/article/10.3390/ijms222312629/s1.
